# Partnerships supporting policies: A social marketing case study of mask supply solutions in South Korea during the COVID-19 pandemic

**DOI:** 10.3389/fpubh.2022.1065310

**Published:** 2023-01-10

**Authors:** Hye-Jin Paek, Thomas Hove

**Affiliations:** Department of Advertising and Public Relations, Hanyang University, Ansan, Republic of Korea

**Keywords:** social marketing, 6 P's, masks, COVID-19, public-private partnership, government regulation, information communication technology (ICT)

## Abstract

**Background:**

South Korea was one of the first countries to experience a large outbreak of COVID-19. Early on, public health authorities recommended mask wearing as one of the main preventive measures against the virus. Compared to people in other countries, most South Koreans were willing to follow this recommendation. However, during the early stages of the pandemic, panic buying and vendor hoarding led to a nationwide mask shortage. The problem that needed to be solved was not to change the public's behavior but rather to make masks more available to them.

**Case description:**

To stabilize mask supply and demand, the South Korean government implemented a distribution system that limited mask purchases to two per person per week, in a 5-day rotation determined by birth year. The places designated for selling masks included pharmacies, post offices, and marts that had access to data about registered South Korea residents who had and had not bought their allotted masks. Despite this system, supply problems persisted. In different regions of the country, population density and demand varied, and some pharmacies sold out their stocks of masks earlier than others. Recognizing that people needed a more effective system for finding masks, the government made mask inventory data available to companies and the general public. Three weeks later, about 200 mask apps and web services had been launched. Supplies of masks quickly stabilized, and people could more easily find and buy them. In addition, pharmacies were able to sell out their stocks of masks more efficiently.

**Conclusion:**

The South Korean case of mask demand and distribution during COVID-19 illustrates how all six of the social marketing components (policy, supplemented by products, price, place, promotion, and partnerships) need to be coordinated for effective mitigation of infectious disease. In particular, this case highlights the importance of public-private partnerships among the government, production companies, retailers, and members of the general public.

## Introduction

During the early weeks of the COVID-19 pandemic, South Korea experienced a severe shortage of health masks needed to curb the spread of the virus. As the population's demand for masks skyrocketed, prices also surged because manufacturers lacked sufficient production capacity to keep up with the sudden population-wide use of masks. The shortage was aggravated by panic buying and hoarding of masks, as well as by efforts among vendors to maximize profits and corner the mask market. One year before the COVID-19 outbreak, in February 2019, the average price for one mask in a bulk purchase ranged from 500 to 800 Korean won (KRW; about 0.45–0.70 USD). In February 2020, that price increased more than tenfold to 10,000 KRW (1). This situation led to increased news coverage of the mask shortage and widespread criticism of the government's apparent lack of action on the issue (2).

To deal with this crisis, the government took urgent measures and enacted a series of policies aimed at increasing the availability of masks in the domestic market. However, coercive policies mandating behavior change among producers and vendors were not in themselves sufficient for making masks available and accessible. The government also launched a variety of public-private partnerships that eventually helped to resolve the mask supply crisis. This case study aims to illustrate how all six of the social marketing components (*policy*, supplemented by *products, price, place, promotion*, and *partnerships*) need to be coordinated during efforts to solve a product shortage during a public health crisis. The goal of our analysis is to suggest how future efforts to meet public demand for scarce health products can use the latter five components to enhance the effectiveness of government policy measures.

## Background

On January 6, 2020, the Korean Associated Press (Yonhap News Agency) reported that 59 patients had contracted an unknown strain of pneumonia in Wuhan, China. These were the first reports about an infectious disease that the World Health Organization (WHO) declared to be COVID-19 on March 11, 2020 (3). The first case of the virus in South Korea was documented on January 20, 2020, during the Lunar New Year holidays. Soon after, South Korea became one of the first countries to experience a large outbreak of COVID-19. The disease would become a worldwide pandemic infecting more than 650 million people, resulting in about 6.6 million deaths as of December 2022, and having no clear end in sight. In South Korea alone, more than two thirds of the Korean population became infected (about 28 million people) with more than 30 thousand deaths (4).

In the early weeks of the COVID-19 outbreak, Korean public health authorities recommended preventive actions such as hand washing, covering the mouth when coughing, and wearing masks. As in other countries, mask wearing was recommended mainly for people with respiratory symptoms visiting health clinics. In the US., for example, the Surgeon General had initially recommended that the general public not wear face masks. However, as researchers gradually confirmed that the virus spreads through aerosols and respirated droplets, the U.S. Centers for Disease Control (USCDC) recommended that people in public settings should at least wear cloth face coverings, if not surgical masks or N95 respirators (5). The World Health Organization also recommended that surgical masks should be worn only by healthcare workers, or by infected people, or by caretakers of infected people (6). Constantly evolving research findings would lead to other changes in mask-wearing recommendations. For example, an observational study showed that using face masks was 79% effective in preventing COVID-19 transmission if people used them before symptoms appeared (7). An evidence review also concluded that mask wearing recommendations and mandates were most effective at reducing spread of the virus when compliance was high (8). However, the same study also pointed out some drawbacks of mask wearing, including the following: risk compensation behavior among the general public (that is, neglecting other preventive behaviors such as hand washing and physical distancing); difficulties in managing supply chains for N95 respirators and surgical masks; and government reliance on coercive policies for securing compliance.

While mask shortages were a global phenomenon, public compliance with mask wearing varied from country to country. According to an April 2020 survey spanning 15 major countries, the countries with the highest rates of mask use were Vietnam (91%), China (83%), Italy (81%), Japan (77%), and India (76%) (9). By contrast, in other countries — particularly western regions such as the United States, Europe, and Russia — rates of mask use were below 54% (10). South Korea was an exceptional case with respect to mask supply and rates of mask use among the general population. According to a July 2020 public opinion survey, more than 90% of South Korean residents agreed that they should wear masks in indoor public places such as public transportation and retail stores, and about 76% agreed that they should wear masks in outdoor multi-use facilities (11).

As of 2014, the Korean Ministry of Food and Drug Safety designates two types of masks that require official testing for quality control — surgical masks and health masks. The specific purpose of health masks is to protect respiratory organs from microbial infections and harmful particulate substances such as atmospheric yellow dust and fine dust caused by pollution. Before the 2020 pandemic, South Koreans would often wear health masks on days when there were serious warnings about fine dust air pollution (12). According to a survey conducted in South Korea in 2019, around 53 percent of respondents stated that they wore masks when a fine dust alert was issued, an increase from 29% in 2014 (13). Up to the outbreak of COVID-19, there had never been a shortage of health masks in normal circumstances, nor even during the increasingly common periods of bad air pollution.

## The mask shortage: Policy and social marketing solutions

During COVID-19, a key goal for public health authorities was to influence people to adopt behaviors that would help prevent or at least curb the spread of the virus. One of the most important preventive behaviors was wearing masks (7, 8). Although Koreans were willing to wear masks (12), domestic supplies were insufficient. To make masks more available, public health authorities enacted several regulatory policies. Although these policies did lead to increased mask production, other social influence strategies would be needed to maximize mask wearing rates among South Korean residents.

One theoretical perspective that sheds light on this situation is the MOA framework. The acronym stands for the variables of motivation, opportunity, and ability. Motivation is defined as goal-directed arousal. Opportunities refer to circumstances in which people have easy access to resources and products. Ability refers to individual skill or proficiency at solving problems. The MOA framework is based on a theoretical model of how consumers cognitively process advertising messages based on their levels of each variable (14). Rothschild (15) extended the MOA framework to a broader range of situations in which the goal is to induce behavior changes. He deduced a variety of circumstances in which those changes are more or less likely to be achieved by three influence strategies: education, policies, and social marketing. Rothschild proposed that education, which entails informing and persuading, is most likely to influence people to change their behaviors when they are already motivated and have adequate opportunities to do so. By contrast, coercive policies and laws would be more appropriate in situations where people are unmotivated, or even resistant, to adopting desired behaviors, and when opportunities to perform them are scarce. Social marketing — the strategy of influencing people to change their behaviors by offering them something desirable in exchange — is most likely to work in situations defined by scarce opportunities yet high motivations.

During the COVID-19 pandemic, various social marketing strategies were used to reduce the spread of the virus (16). The behavior change methods distinctive to social marketing are, first, to develop tangible products that can facilitate behavior changes and, second, to make those products cheap, accessible, and available in various prosocial and health contexts (17–19). Much of the South Korean population was motivated to buy and wear masks, but opportunities to do so were scarce because of the mask shortage. To solve that problem, the government initially intervened with legal policies that mandated increased production of masks and regulated where and when people could buy them. However, these policy solutions would eventually need to be supported by educational and social marketing strategies. Regarding education, much of the public still needed to be informed or reminded about the proper use of masks. As for social marketing, a proper mix of the classic 5Ps of marketing — product, price, place, promotion, and partnerships — supplemented the coercive policy efforts to make masks more available (see Table 1).

**Table 1:** Social marketing components.

**Six Ps**	**Concept definition**	**Application to the current case**
**Policy**	A law, regulation, procedure, or administrative action of government (22).	➢ Emergency Supply and Demand Measures for Health Masks (ESDMHM). ➢ Price Stabilization Act. ➢ Five-Day Mask Distribution System.
**Product**	Core behavior.	➢ Prevent the spread of COVID 19 to save lives.
	Actual behavior.	➢ Mask wearing.
	Augmented behavior.	➢ Health masks.
**Price**	Monetary and non-monetary costs.	➢ Monetary: 1,500 KRW per mask. ➢ Non-monetary: Time and efforts needed to find and buy masks (relieved by apps).
**Place**	Where and when the target market will buy the augmented behavior.	➢ Public vendors: pharmacies, post offices, the National Agricultural Cooperative Federation (Nonghyup).
**Promotion**	Communication, message, and media strategies for creating awareness about and positive attitudes toward the product.	➢ Communication strategy: use credible spokespersons. ➢ Message strategy: convey factual information, accompanied by expressions of empathy (thanking the public for their cooperation, expressing regret for ongoing problems, promising solutions). ➢ Media strategy: provide regular briefings and updates on TV, social media, posters and fliers, SMS text alert messages, mask location apps and services.
Partnership	An arrangement in which parties agree to cooperate to advance their mutual interests.	➢ Within the government: enact and execute the policy, use partnerships to develop the mask app (MSF, MHW, MFDS, KCDC, MCST, and MSICT). ➢ In the public sector: provide and use data on mask availability (HIRAS and NIA). ➢ In the private sector: cooperate as public vendor (KPA); develop mask apps (KT, NHN, Koscom, NBP, Naver, Kakao, and civic hackers).

MSF, Ministry of Strategy and Finance; MHW, Ministry of Health and Welfare; MCST, Ministry of Culture, Sports and Tourism; MSICT, Ministry of Science and ICT; HIRAS, Health Insurance Review and Assessment Service; NIA, National Intelligence and Information Society Agency; KPA, Korean Pharmaceutical Association.

### Government policy measures

The main targets of government policy measures were mask manufacturers and vendors. On February 5, 2020 the government, fighting efforts to corner the mask market, enacted a prohibition notice banning vendors from possessing and hoarding excessive stocks of masks. On February 12, the first Emergency Supply and Demand Measures for Health Masks (hereafter, ESDMHM) were enacted. These measures required mask producers and vendors to report production output, sales volume, sales prices, and sales destinations. On February 26, as the daily rates of COVID-19 infections were rising, the initial ESDMHM were revised to include reporting requirements for not only health masks but also surgical masks. To make mask supplies more available to the general public, the measures required all mask producers to release 50% of their production output to retailers, pharmacies, post offices, and marts run by the National Agricultural Cooperative Federation (hereafter, “Nonghyup marts”). Exports of masks to other countries were also restricted. For example, the total number of masks set aside for export could not exceed more than 10% of a producer's daily yields (1). During these initial weeks, the government held back from directly regulating mask prices because the quality and cost of masks varied by manufacturer, and because the surge in demand led to increased costs of raw materials needed for production.

Despite these policy measures applied to mask producers and vendors, the supply and demand of masks failed to stabilize. Whenever masks were available for online purchasing, they sold out instantly, even if they were exorbitantly priced. When masks were available to buy in brick-and-mortar, big-box, and department stores, large crowds would rush to buy them. Photos and videos of long, snaking lines of people with anxious faces became media spectacles and intensified public attention to the mask shortage. According to a content analysis of news frames in South Korea, the second stage of COVID-19 in 2020 (February 19–29) was defined by the news media's focus on the chaos surrounding mask supplies (2). The same study found that newspapers — particularly more progressive ones — published many stories on the mask supply problem and the possible solutions for it.

As the supply problem continued, on March 6 the government tried to stabilize mask prices and facilitate equitable distribution among the general public by enacting the third and strongest version of the ESDMHM. This version added the following regulations for mask producers. Overseas exports of masks were categorically prohibited. From each day's production yield of masks, 80% had to be released to vendors on the same day. Mask producers who wanted to sell more than the supplies reserved for pharmacies, post offices, and Nonghyup marts could do so only with government permission. In addition, the government encouraged mask producers to operate their factories at full capacity on weekends. Between February 25 and April 22, 2020 the Ministry of Food and Drug Safety (MFDS) — the agency mainly in charge of licensing, testing, and managing mask supplies during the COVID-19 outbreak — sent groups of staffers in regular rotations to the mask production facilities of the companies that had been approved and registered to manufacture masks (20). The purpose of these visits was to assist mask producers in a variety of tasks, including packaging masks, loading boxes, and completing paperwork. Another policy, the Price Stabilization Act, set a limit for the highest price at which masks could be sold. Some producers, particularly those who made high-quality masks, protested that the government's volume and price control requirements were unreasonable. However, a compromise was eventually achieved.

Other regulations from the third version of the ESDMHM applied to mask vendors and retailers. The government mandated a standard price of 1,500 KRW per mask. In pharmacies, post offices, and Nonghyup marts, customer purchases were limited to two masks per person once every week in a 5-day rotation determined by the final number of a person's birth year (e.g., 1 and 6 on Mondays, 2 and 7 on Tuesdays, and so on; missed purchase opportunities could be rectified on weekends) (21). To enforce this limit, pharmacies and the other designated vendors were temporarily allowed to collect, store, and use customers' personal information, including their resident registration numbers and purchase history.

### Product

In social marketing, the concept of the product has three dimensions: the core product, the actual product, and the augmented product (22). The actual product is the desired behaviors that social marketing campaigns aim to promote. The core product includes the benefits associated with those behaviors. The augmented product consists of the tangible goods and services that enable people to adopt those behaviors. In COVID-19 mitigation efforts, preventive behaviors such as mask wearing constitute the actual product. The core product is the benefits of those behaviors, for example preventing the spread of COVID-19 and saving lives. For the specific behavior of mask wearing, the augmented product is the masks themselves.

### Price

The concept of price in social marketing includes both the monetary and non-monetary costs that the target population associates with adopting the desired behavior (22). People are more likely to adopt a behavior if they perceive its benefits to outweigh its costs, and less likely to adopt it if they perceive the costs to have more weight. During the government's enforcement of the mask distribution system, monetary costs remained similar for everyone because the government mandated a fixed price. The major price issues were related to non-monetary costs such as the time, effort, and energy required to find masks when they were in short supply and available for purchase only once a week on a specific day for each person. In these circumstances, the challenge was to make masks more accessible for ease of purchase. Accordingly, place and promotion strategy played important roles.

### Place

During the mask shortage, the most important place-related variables were where and when the target population could buy masks. As part of their efforts to regulate mask supply and distribution, the South Korean government designated pharmacies, post offices, and Nonghyup marts as licensed mask retailers. According to statistics from the Health Insurance Review and Assessment Service, in 2020 there were 23,305 pharmacies in South Korea (23). The country's number of pharmacies per 100,000 people was 1.4 times higher than the OECD average (24). During the early weeks of the pandemic, even though residents of urban areas could easily find nearby pharmacies, few had masks in stock. From a social marketing perspective, the key challenge was to help people find masks as easily as possible.

In efforts to meet that challenge, smartphones and the Internet would play important roles. According to the OECD, as of 2020 South Korea's Internet usage rate was 96.5% (25). Among South Korean adults, the mobile phone usage rate was 93% (26). This high usage rate suggested that smartphones and the Internet could be used in conjunction with geo mapping technology to inform people about the locations of pharmacies with masks in stock. Since geo mapping technology was more advanced in private sector businesses, public-private partnerships were crucial for facilitating mask distribution. Such partnerships will be described in more detail below.

### Promotion

In social marketing, the purposes of promotion are, first, to make people aware of the desired behavior and, second, to influence them to have a positive attitude toward it. Promotion has several components: the messages that need to be communicated; sources who can communicate the messages credibly and effectively; communication channels and media platforms on which the messages can be disseminated; and proper timing and placement of the messages. During the mask shortage, there was relatively little need for messages about the benefits of wearing masks. Public health authorities and news media had already adequately disseminated that message, and most people were already willing to wear masks (12). Instead, messages were needed to inform people about how the 5-day mask distribution system worked, and how they could locate pharmacies with masks in stock.

Credible message sources and channels had already been established. For several weeks after January 20, 2020, when the first domestic COVID-19 patient was confirmed, the directors of the KCDC and the Central Disaster Management Headquarters (part of the Ministry of Health and Welfare, or MHW) regularly held two media briefings per day. A morning briefing covered the government's COVID-19 response and management strategies, and an afternoon briefing provided updates about confirmed cases, policy measures, and epidemiological research findings (27). Public opinion surveys consistently showed that people attributed high credibility to these two public health agencies. For example, in July 2020, several months into the pandemic, people reported continuing high levels of trust: up to 90% for the KCDC, and up to 75% for the MHW (28).

In addition, during the mask shortage period, the head of the Ministry of Food and Drug Safety (MFDS) offered daily briefings that specified daily mask production rates, reminded people about the final birth-year digits of people who could buy masks on the current day, and covered any other new mask-related policies and updates. This information was also provided on the social media feeds and websites of various national and local government agencies. During the 1st week of the 5-day mask distribution system, the daily birth-year digit information related to mask buying eligibility was sent *via* emergency disaster text messages temporarily under the name of MFDS. These and other details about the 5-day mask distribution system were also communicated on fliers and posters that the government sent to pharmacies, post offices, and Nonghyup marts.

### Partnerships

Public-private partnerships played especially important roles in the five-day mask distribution system. Several government agencies formed partnerships with different types of businesses. The Ministry of Food and Drug Safety was in charge of working with pharmacies and managing mask supplies and safety. The Ministry of Culture, Sports, and Tourism assisted in efforts to educate and inform the public. This ministry's duties include promoting government policies, as well as managing and evaluating the public relations and communication activities of various government agencies. They accordingly helped develop messages and communication strategies across various media platforms that could effectively inform the public about the mask distribution system. The Ministry of Strategy and Finance was in charge of applying and executing the Price Stabilization Act. The Ministry of Science and ICT managed mask distribution for post offices and established other partnerships for developing websites and apps that informed people about mask supplies and buying eligibility.

To make the mask distribution system work, pharmacists had to carry out several additional duties. They re-sorted mask supplies so that masks could be sold in sets of two. They entered customers' resident registration numbers into the computerized system that monitored national mask purchases. In situations when customers wanted to buy more than their allotted share, or when they claimed they were buying masks on behalf of others, pharmacists had to deal with their complaints. To ease these extra burdens, officials from the federal and local governments were sent to help pharmacists check ID cards, sell masks, distribute promotional materials, and deal with civic complaints and disputes related to the mask shortage.

Although the government made efforts to disseminate information widely about who could buy masks and when, people also needed up-to-date communication about where masks were still in stock. Real-time tracking systems informed by geo mapped data were urgently needed because the population density and demand for masks varied from region to region, and because pharmacies in different regions sold out their mask stocks at different rates. Recognizing that people needed a more effective system for finding masks, the government made mask inventory data available to companies and the general public.

The procedure was as follows. The Health Insurance Review and Assessment Service (HIRAS) provided a system that could operate on pharmacy and post office computers that recorded the identification numbers of customers making mask purchases and checked for duplicate purchases. This system then provided data such as sales destinations and sales status to the National Information Agency (NIA). NIA reprocessed those data and made them available in an open application programming interface (API). Companies with cloud computing infrastructure (e.g., KT, NHN, Koscom, and NBP) provided free storage and other resources for “civic hackers” to develop apps that would enable the general public to access updated information about mask supplies. In addition, Internet companies such as Naver and Kakao allowed developers to use their APIs free of charge (29).

This elaborate public-private partnership network enabled civic hackers to develop several apps that, after some trial and error, provided the general public with accurate and timely data on mask availability. Initially there were 17 app developers, but the number eventually went as high as 170. All of them were unpaid volunteers. After a 5-day testing period in which small amounts of data were available to use for troubleshooting, the government made the complete raw data available on March 10, 2020. The first beta mask app service launched on the next day, but it had several problems related to high user volume, Internet congestion, system overloads, slow connections, and lags and discrepancies in the real-time information about mask supplies. As a result, people still had difficulty locating and buying masks. Taking rapid action to solve these problems, the government installed additional web servers to increase the maximum number of simultaneous users that the system could accommodate. Efforts were also made to improve the apps' accessibility features for people with hearing and vision deficiencies (30). In the course of 3 weeks, about 200 mask apps and web services had been launched. Supplies of masks quickly stabilized, and people could more easily find and buy them.

### Evaluation of the mask distribution program

The government's regulatory efforts to increase mask supplies were ultimately successful. By June 2020, the daily supply of masks increased enough to render most of the ESDMHM regulations unnecessary. According to a whitepaper published by the Ministry of Food and Drug Safety (20), the number of mask production companies increased from 197 in January 2020 to 1,591 in the 1^st^ week of July. The number of products officially licensed as health masks quadrupled, from 953 on January 1, 2020 to 3,607 in the 1^st^ week of July, 2020. The weekly health mask production capacity increased from 46,130,000 in January 2020 to 106,530,000 in the fourth week of June, 2020. The number of masks in stock increased 100-fold from 11,260,000 on February 2020 to 1,152,260,000 on March 2021. During the 5-day mask distribution system between the 2^nd^ week of March and the 1^st^ week of July, a total of 951,950,000 masks were supplied.

Despite some initial problems, public evaluation of the mask distribution system was generally positive. During the 1^st^ week of the system's implementation, 59% of people surveyed claimed that buying masks was difficult, and only 23% claimed it was easy. Three weeks after the mask supply apps were launched, those proportions changed from 40% (difficult) to 48% (easy) (31). The mask supply app services also aided pharmacy sales. Before the apps were released, only 39.5% of pharmacies had sold out their stocks of masks. After the apps were released, that number grew to 92.5% (32). According to public opinion polls conducted by *Korea Research*, about 68% of the public approved of the 5-day mask distribution system (33). Polls also indicated that the system received increasingly positive evaluations after the mask supply apps were available for public use. Another public opinion survey conducted by a different company (*Embrain Public*) on March 15, 2020, reported that 64.1% of the survey respondents (*N* = 1,005) had positive evaluations of the 5-day mask distribution system (34).

As of July 11, 2020, the government abolished the purchasing limit of two masks per person on designated days, allowing people to buy masks at pharmacies, post offices, and Nonghyup marts in larger quantities on any day of the week. On July 12, the public supply system for health masks was curtailed, and regulations on market supply-and-demand transactions were loosened (20).

## Discussion

As proposed in Rothschild's (15) MOA framework, efforts to influence people to adopt desired behaviors require different strategies in different circumstances. Education — particularly in the form of informative media campaigns — may be a sufficiently effective public health influence strategy when people are already motivated to change their behavior and have opportunities to do so. But when people are not motivated to perform desired behaviors, coercive policies may be a more effective strategy. The case of the South Korean mask shortage suggests that, in the latter circumstances, coercive policies may need the additional support of education and social marketing. In the early stages of the COVID-19 pandemic, government intervention in the marketplace was needed to prevent producers and vendors from cornering the mask market, and to prevent consumers from hoarding masks. The Emergency Supply and Demand Measures for Health Masks (ESDMHM) and the 5-day mask distribution system were implemented to make mask buying opportunities more equitable among the national population. Although the 5-day mask distribution system seemed to be a sensible policy on paper, in its early stages it did not run smoothly. Other strategies would be needed to meet the urgent public demand for masks and make buying them easier and more convenient.

This case of the South Korean mask shortage illustrates how coercive policy measures — the strictest tools for influencing behavior — can become more effective with additional support from social marketing strategies. Social marketing techniques helped achieve the following goals with respect to the following four components: increase the desired behavior of mask wearing (product); reduce people's non-monetary costs such as the time and effort necessary for buying masks (price); provide real-time information about pharmacies, post offices, and Nonghyup marts where people could buy masks (place); and keep people regularly informed about available mask supplies and the eligibility requirements for buying them (promotion).

The most important social marketing component of this case was partnership. The network of partnerships between government agencies and private companies helped increased mask supplies and made them more easily and equally accessible to the general public. There were several coordinated efforts among multiple government agencies to achieve these goals, and those efforts were aided by the cooperation of private entities such as mask producers, the Korean Pharmaceutical Association, civic hackers, and ordinary citizens.

These partnerships had tensions, but those tensions were ultimately resolved. Mask producers initially resented stricter government control over their production, pricing, and selling practices. However, they eventually realized that having the government buy most of their production output at a fixed price would yield them more revenue than they would have earned going through ordinary competitive market negotiations with vendors. Pharmacists felt burdened by additional duties related to repackaging masks and recording customer and supply information, and they became stressed out dealing with public complaints about mask-buying restrictions. However, these burdens were partly relieved by the apps that emerged from the partnerships among government agencies and civic hackers. Moreover, those apps reduced some of the pharmacists' workload and enabled them to sell their stocks of masks more effectively. Among the general public, people initially complained about the limited quantities and high prices of masks. However, they became accustomed to the 5-day distribution system because it solved problems such as waiting in long lines and being uncertain where and whether one could buy masks.

Several other issues should be noted. First, even in exceptional situations like the COVID-19 pandemic, it is unusual and risky for governments to intervene so directly in the marketplace by controlling product supplies and prices. While many stakeholders cooperated with this intervention, its possible long-term effects are not yet known. For example, there has been an exponential increase in the numbers of mask manufacturers and masks produced. Whenever COVID-19 attenuates and people are no longer required to wear masks, the market might experience turmoil, and many manufacturers might face bankruptcy. Second, people's privacy may have been violated by the recording of personal data required for the 5-day mask distribution system to work. People had to supply their personal identification (resident registration) numbers to pharmacists, and then pharmacists entered that information into the nationwide system that monitored mask purchases. However, those data were made publicly available so that the mask app services could be developed. This is one of those situations in which debates are likely to arise about the relative priorities of individual rights and public safety. The Korean government passed the Infectious Disease Control and Prevention Act (IDCPA) to establish a legal basis for collecting and disclosing patient information. However, people continue to disagree about how much information would be acceptable or appropriate to disclose (35). Notwithstanding these legal and privacy issues, residents of South Korea were generally willing to comply with the government's public safety measures and recommendations, particularly mask wearing. But of course, the public's toleration of government interventions and directives is likely to vary from one country to another.

While this case illustrates how government policy measures can be more effective when aided by the other 5Ps of social marketing, some limitations should be noted. First, the government's message promotion strategies need more rigorous scrutiny. Although the government made serious efforts to provide consistent messages from credible sources *via* multiple media channels, more research is needed to determine what would be the most effective kinds of message strategies (e.g., in terms of message tone, frames, formats, and narratives). Moreover, the flood of messages pouring out from the press and various government agencies may have given people information fatigue. For example, a recent survey study among 821 South Korean adults reported that COVID-19 message fatigue was positively related to information avoidance and heuristic processing, which in turn led to greater levels of misinformation acceptance (36). A second limitation is that better data are needed for evaluating the effectiveness of the government's measures for improving mask supply and distribution. A series of public opinion surveys, along with several government whitepapers written for purposes of record-keeping, have been useful tools for evaluating the short-term effectiveness of those measures. However, these documents rely on secondary data, whereas primary data would provide better ways of assessing the effectiveness of the various policy and social marketing measures. In addition, data are currently lacking for assessing the possible long-term effects of the mask supply and distribution programs, particularly on outcomes such as mitigating the spread of COVID-19 and saving lives.

Despite these limitations, this case study suggests lessons for other national-level situations in which an essential health product becomes scarce during a public health crisis. For example, South Korea had to deal with a similar shortage of self test kits after the Omicron variant of COVID-19 spread through the country during the early months of 2022 (37). Applying lessons from the 5-day mask distribution system, the government enacted similar policies to increase supplies and regulate purchases of self test kits. For example, online sales were banned, purchases were limited to five per person, and availability was enhanced by adding convenience stores to the list of designated vendors (38). This type of nationwide product shortage during a public health crisis should not happen too often. However, whenever such problems happen again, the South Korea mask shortage case illustrates how government policies can be made more effective by social marketing strategies. The essential elements in both of these cases are the following: well-designed policy measures that are broadly, equitably, and effectively enforced; cooperative partnerships among multiple government agencies and a wide variety of private companies, organizations, and groups; and information communication technology that facilitates the distribution and purchasing of the scarce product.

## Conclusion

A variety of social marketing principles and practices were used in efforts around the globe to mitigate the spread of COVID-19 (16). One of the limitations of social marketing as a strategy for influencing people's behaviors is that it ultimately relies on voluntary choice. The urgent circumstances of a worldwide pandemic would also require some degree of coercive government and legal policies that could be enacted quickly and executed effectively. In addition, while public-private partnerships are often neglected in social marketing literature, they are crucial for establishing networks of cooperation that can make policy and social marketing strategies more effective. This case study suggests how supplementing policy measures with social marketing strategies — particularly partnerships — can help overcome a major obstacle in efforts to deal with a public health crisis.

## Data availability statement

The original contributions presented in this study are included in the article/supplementary material. Further inquiries can be directed to the corresponding author.

## Author contributions

H-JP compiled and wrote up details and information from South Korean sources about this case. TH compiled and integrated theories and information from relevant non-Korean literature. Both authors developed the argument and wrote the text in close and constant collaboration in a shared file.

## References

[1] Kim Y. Regulatory methods and countermeasures for surging mask prices and cornering the market. Issue Paper 20-02. Korea Legislation Research Institute. (2020).

[2] Park J-H. A comparative study on the ‘Corona19' news frame based on ideological orientation of media. Korean J Journalism Commun Stud. (2020) 64:40–85. 10.20879/kjjcs.2020.64.4.002

[3] Lee D. Entry of about 1800 people from Wuhan, China … “MERS-level response.” Hankook Ilbo. (2020). Available online at: https://www.hankookilbo.com/News/Read/202001061378071086?t=20220907162712 (accessed October 7, 2022).

[4] CoronaBoard. COVID-19 live situation board. (2022). Available online at: https://coronaboard.kr/ (accessed December 18, 2022).

[5] Meredith S. In a U-turn, US surgeon general asks CDC to see if face masks can prevent coronavirus spread after all. CNBC. (2020). Available online at: https://www.cnbc.com/2020/04/02/coronavirus-us-surgeon-general-asks-cdc-to-see-if-face-masks-are-effective.html (accessed October 7, 2022).

[6] Chan AL Leung CC Lam TH Cheng KK. To wear or not to wear: WHO's confusing guidance on masks in the covid-19 pandemic. The BMJ. (2020). Available online at: https://blogs.bmj.com/bmj/2020/03/11/whos-confusing-guidance-masks-covid-19-epidemic/ (accessed October 9, 2022).

[7] Wang Q Yu C. The role of masks and respirator protection against SARS-CoV-2. Infect Control Hosp Epidemiol. (2020) 41:746–7. 10.1017/ice.2020.83 32192550 PMC7139364

[8] Howard J Huang A Li Z Tufekci Z Zdimal V van der Westhuizen H-M . An evidence review of face masks against COVID-19. Proc Nat Acad Sci. (2021) 118:e2014564118. 10.1073/pnas.2014564118 33431650 PMC7848583

[9] Ipsos. More people say they're wearing masks to protect themselves from COVID-19 since March. (2020). Available online at: https://www.ipsos.com/en/more-people-say-theyre-wearing-masks-protect-themselves-covid-19-march (accessed October 7, 2022).

[10] Hudaib B Al-shawabkeh AF Hudaib F. Knowledge and awareness of masks and N95 Respirators used for COVID-19 prevention among chemical engineering students at Al-Balqa Applied University, Jordan. Front. Public Health. (2022) 9:620725. 10.3389/fpubh.2021.620725 35071146 PMC8781538

[11] Lee DH. [COVID-19] Social Psychology of Wearing Masks-Why People Wear Masks. Regular Survey of Public Opinion. Korea Research. (2020). Available online at: https://hrcopinion.co.kr/archives/15994 (accessed October 9, 2022).

[12] Kang J Jang YY Kim J Han SH Lee KR Kim M . South Korea's responses to stop the COVID-19 pandemic. Am J Infect Control. (2020) 48:1080–6. 10.1016/j.ajic.2020.06.003 32522606 PMC7834720

[13] Statista Research Department. Share of people wearing masks when a fine dust alert issued South Korea 2014-2019. (2022). Available online at: https://www.statista.com/statistics/1047573/south-korea-wearing-masks-when-a-fine-dust-alert-issued/ (accessed December 18, 2022).

[14] MacInnis DJ Moorman C Jaworski BJ. Enhancing and measuring consumers' motivation, opportunity, and ability to process brand information from ads. J Mark. (1991) 55:32–53. 10.1177/002224299105500403

[15] Rothschild ML. Carrots, sticks, and promises: A conceptual framework for the management of public health and social issue behaviors. J Mark. (1999) 63:24–37. 10.1177/002224299906300404

[16] Lee NR. Reducing the spread of COVID-19: A social marketing perspective. Soc Mar Q. (2020) 26:259–65. 10.1177/1524500420933789

[17] Gordon R McDermott L Stead M Angus K. The effectiveness of social marketing interventions for health improvement: What's the evidence? Public Health. (2006) 120:1133–9. 10.1016/j.puhe.2006.10.008 17095026

[18] Olson DJ Purdy C Harrington R Marun D Garcia JE. How social marketing contributed to expanding size of overall condom markets in Ethiopia, Brazil, and Indonesia. Soc Mar Q. (2022) 28:91–108. 10.1177/15245004221077965

[19] Stead M Gordon R Angus K McDermott L. A systematic review of social marketing effectiveness. Health Educ. (2007) 107:126–91. 10.1108/09654280710731548

[20] Ministry of Food and Drug Safety. The 2021 COVID-19 response in the Ministry of Food and Drug Safety. Whitepaper. (2021).

[21] Kim H. Lesson learned from the power of open data: Resolving the mask shortage problem caused by COVID-19 in South Korea. Sustainability. (2021) 13:278. 10.3390/su13010278

[22] Lee NR Kotler P. Social Marketing: Influencing Behaviors for Good. Thousand Oaks, CA: Sage Publications (2011).

[23] Kim MG Jeong HJ. The five-day mask rotation system worked...Government-distribution-pharmacy joint work. DailyPharm. (2020). Available online at: http://www.dailypharm.com/Users/News/NewsView.html?ID=263633 (accessed October 9, 2022).

[24] Kang SK. The number of pharmacies per 100,000 people is 1.4 times higher than the OECD average. DailyPharm. (2020). Available online at: http://www.dailypharm.com/Users/News/NewsView.html?ID=264559 (accessed October 9, 2022).

[25] KOSIS. Topical statistics: National Internet usage rates. (2022). Available online at: https://kosis.kr/statHtml/statHtml.do?orgId=101andtblId=DT_2KAAA13_OECD (accessed October 7, 2022).

[26] Gallup Research Center of Korea. 2012-2021 Smartphone usage and brand, smartwatch, and wireless earphones survey. (2021). Available online at: https://www.gallup.co.kr/gallupdb/reportContent.asp?seqNo=1217 (accessed October 7, 2022).

[27] Paek H-J Hove T. Communicating uncertainties during the COVID-19 outbreak. Health Commun. (2020) 35:1729–31. 10.1080/10410236.2020.1838092 33198519

[28] Lee SJ. [COVID-19] 12th perception survey (Situation Perception, Public Subject Reliability, etc.). Korea Research. (2020). Available online at: https://hrcopinion.co.kr/archives/16099 (accessed October 9, 2022).

[29] Lee JY Lee JH Kim YH. A study on the e-Governance Network in the development process of public mask applications for COVID-19. Informatization Policy. (2021) 28:23–48. 10.22693/NIAIP.2021.28.3.023

[30] Ministry of Science and ICT. A mask app, a complete body of collective intelligence created by the government and the private sector. (2020). Available online at: https://blog.naver.com/with_msip/222123296473 (accessed October 9, 2022).

[31] Lee DH. [COVID-19] 4th perception survey (Situation Awareness, Social Distancing, etc.). Korea Research. (2020). Available online at: https://hrcopinion.co.kr/archives/15262 (accessed October 9, 2022).

[32] Ministry of Science and ICT. This combination, very successful! Government and civic hacker “public mask app”. (2020). Available online at: https://blog.naver.com/with_msip/222121007408 (accessed October 8, 2022).

[33] Lee DH. [COVID-19] 3rd perception survey (Related Policy Evaluation, five-day mask system, etc.). Korea Research. (2020). Available online at: https://hrcopinion.co.kr/archives/15179 (accessed October 9, 2022).

[34] Donga I. “Five-day mask system” is 64% positive… The government responds well, rising to 61 percent. (2020). Available online at: https://www.donga.com/news/Society/article/all/20200315/100162879/1 (accessed December 18, 2022).

[35] Paek H-J Hove T. Information communication technologies (ICTs), crisis communication principles and the COVID-19 response in South Korea. J Creat Commun. (2021) 16:213–21. 10.1177/0973258620981170

[36] Hwang Y So J Jeong SH. Does COVID-19 message fatigue lead to misinformation acceptance? An extension of the risk information seeking and processing model. Health Commun. (2022) 1–8. 10.1080/10410236.2022.2111636 35968837

[37] Kim YW Kim YM Lee SM Lim JH Lim DS Park YJ. Analysis of the occurrence status and related factors of COVID-19 confirmed patients and cohabitants during the period of omicron dominance. Weekly Health Dis. (2022) 15:951–955.

[38] Ministry of Public Health and Welfare. In preparation for the omicron epidemic, from February 3rd (Thu), gradual change to the examination and treatment system involving local hospitals and clinics. Regular briefings. (2022). Available online at: https://www.mohw.go.kr/react/al/sal0301vw.jsp?PAR_MENU_ID=04&MENU_ID=0403&page=1&CONT_SEQ=370004

